# Are we ready to build health systems that consider the climate?

**DOI:** 10.1177/1355819613516943

**Published:** 2014-04

**Authors:** Susannah Mayhew, Sara Van Belle, Michael Hammer

**Affiliations:** 1 Senior Lecturer, Global Health Department, London School of Hygiene & Tropical Medicine, London, UK; 2 Independent Consultant and previously Lecturer, Global Health Department, London School of Hygiene & Tropical Medicine, London, UK; 3 Executive Director, INTRAC, Oxford, UK

**Keywords:** climate change, health systems, sustainable development

## Abstract

At last, climate change does appear to have entered mainstream consciousness. In the scientific community, the climate change debate has shifted from focusing on establishing the truth of the claim that climate change is a reality to warming public opinion to the cause and acknowledging that climate change will have far-reaching effects on how we build, organize and manage climate-responsive social systems including health care systems. There is particular urgency to the debate for health services and systems in low income countries where some of the worst effects of climate change will be felt and where health systems are already over-stretched due to long-term lack of investment, a double burden of disease (preventive and non-communicable), a crisis in human resources and governance deficiencies. Despite the urgency, the health care systems development community appears insular in its interests and actions, and a clear leader that could coordinate the activities of different researchers, research bodies, policy makers and international organizations across relevant sectors including disaster management, climate and health care systems, has yet to emerge. This essay considers the political landscape, possible leaders and why it is necessary for health systems’ professionals to move beyond the health sector in order to secure support for health and health care systems development in a post-Millennium Development Goals development framework that is defined by climate change.

## The challenge of climate change for health care systems

The 2012 Rio+20 summit and the recently concluded 18th Climate Change Summit in Doha reiterated the commitment to developing a timely and ground-breaking new post-Kyoto Protocol, indicating that climate change does finally appear to have irrevocably entered mainstream consciousness.

In the scientific community, the climate change debate has shifted from focusing on establishing the truth of the claim that climate change is a reality to warming public opinion to the cause^1^ and acknowledging that climate change will have far-reaching effects on how we build, organize and manage climate-responsive social systems including health care systems. There is particular urgency to the debate for health services and systems in low-income countries (especially states with particular vulnerabilities to the impact of environmental variability) where some of the worst effects of climate change will be felt and where health care systems are already over-stretched due to long-term lack of investment, a double burden of disease (preventive and non-communicable), a crisis in human resources and governance deficiencies.^2^ Climate change-responsive health care systems must proactively respond to rapid and significant population fluctuations caused by movements of people leaving areas hit by drought or floods, desertification, and damage to traditional crop and livestock farming. Such climate change-triggered migration often results in rapid urbanization.^3^ Changing disease patterns as a result of climate change further increase the need for health systems decision-making to be flexible and able to manage uncertainty, since clinical care and limited resources must be adjusted to reflect evolving population needs.^2^ Other factors including continued population growth and political strife in fragile states may further compound the problem and may further destabilize the response capacity of already weak systems.

## Are health systems practitioners and researchers responding?

In 2010, the WHO established Health Systems Global, which is (in its own words) ‘the first international membership organization fully dedicated to promoting health systems research and knowledge translation’. This represents an extraordinary opportunity to promote thinking on how health systems and service delivery can respond to climate issues, and start defining the goals and challenges that complex health care systems need to respond to. How surprising then that a sense of urgency has been totally missing from its two Global Symposia on Health Systems Research in Montreux in 2010 and in Beijing in 2012, where, as the conference proceedings illustrate, no research was presented on the implications of climate change for health care systems. This apparent lack of interest makes the health care systems research community look insular and out of touch with the current global debate about a post-Millennium Development Goals (MDGs) agenda as well as other related health fields like disaster management. While the exact configuration of the post-2015 development framework remains emergent, it is highly likely that the ‘sustainable development goals’ (for which the process was launched at the Rio+20 conference), which seem likely to be combined with the MDGs successors (though to date the conversations are largely separate), will be game-changing and the place of health and health care systems funding in this context is by no means certain.

The health care research community must contribute to the shaping of the sustainable development research agenda to promote understanding of the implications of climate change on health and health care systems, connecting different levels in the system. There is a renewed focus on complexity in health care systems that means health researchers are well-positioned to join a trans-disciplinary effort with other system researchers to understand how to build adaptive health care systems that co-evolve with other social-ecological systems.

## What should be done?

Kula et al.^4^ highlight a number of issues to be addressed in order to develop climate-resilient health care systems. Adaptive capacity needs to be built and more resilient health care facilities and supply chains developed, considering how these will be affected by extreme climate events (floods, heatwaves, droughts and so on). This includes improving the capacity of health services and human resources to cope with additional disease burdens associated with extreme weather events, including timely provision of medical supplies for increased or changing infectious disease transmission. They also call for a cross-disciplinary research agenda to promote understanding of the health effects of climate change in different settings, linking meteorology, climatology, other relevant sectors and health. This would encourage the development of seasonal forecasting and early warning systems for extreme events affecting health (e.g. heat and flood-health warnings) and for infectious diseases (e.g. epidemic malaria) as well as other health protection surveillance systems for vector- and water-borne diseases. Connections must also be better made to other relevant, well-established bodies of work, particularly in disaster risk reduction, impact mitigation and response. Many of the challenges associated with climate change have resonance with work done over the decades on disaster preparedness and response, and this may be a useful lens with which to consider health systems’ responsiveness to climate change. For example, hazard analysis and vulnerability reduction have long been part of disaster management practice, advocacy and research.^5^ Drawing on the knowledge and experiences of this field could facilitate, for example: the increased response capacity of health systems to absorb migrant populations following climate events (emergency preparedness); improved governance capability to coordinate inter-sectoral action at local level (on health and food security, emergency preparedness) to improve efficiency; and leverage collective action among communities related to disaster and emergency preparedness to protect health. Importantly, action needs to be taken to promote better understanding in both the climate change and public health communities of the ‘health dividend’ of many actions to reduce greenhouse gas emissions: for example, funding alternatives to polluting and respiratory-harming traditional wood/coke-burning cooking stoves. A better understanding of potential mutual benefits would encourage concerted and coherent action to protect health and health care systems in the context of climate change.^4^ So in the cooking stove example, the benefit for the health care system is that less-polluting alternatives may reduce the opportunity cost related to chronic respiratory diseases, freeing up funds spent on curative care for preventive care or for other more pressing health demands.

## Who will lead?

But is the health research community up to this job? The health research community is fragmented and has been slow to take up the challenge of investigating the impacts of climate change on health and health care. Studies are only now emerging to establish the effects of climate change on health outcomes, but findings are still ad hoc and limited.^6–9^ There is virtually no research on the impact of climate change on health systems*,* and there is an urgent need for research on how to strengthen health care systems to be resilient and responsive to the challenges posed by climate change.^4^ Lynch^10^ calls for a defined goal that health care systems actors can coalesce around and is realistic enough to allow ‘players in the health system to realize that climate change is a game changer but not necessarily a game destroyer’ (p. 248). There is much uncertainty also about funding and whether climate funding might draw funding away from regular public health activities. Climate change requires that health care organizations – and health care systems – make new connections and rethink how services are organized, funded and delivered; it may be risky. Who will provide the leadership: the ‘policy and research community’; or institutions such as the World Bank or the WHO?

Currently, there are no clear leaders who could define such a goal. Nevertheless, there are emerging players and initiatives. The Rockefeller Foundation invested over $50 million in a project in Asia specifically addressing the issue of climate change and health (Asian Cities Climate Change Resilience Network). This network is beginning to demonstrate tangible outputs in changing health systems to respond to climate change. The World Bank recognized earlier than many global institutions the importance of climate change for development and is undertaking a re-structuring to make climate issues core to its development work, although the links between health and climate change in its current work remain weak.^11^ The WHO has not shown global leadership on climate change and health, although it has a long history of work on environmental health and disaster management. There has been recent (2008) institutional commitment to working on climate change and health resulting in a World Health Assembly resolution and some slowly emerging briefing papers and reports.^12–15^ Nevertheless, the WHO’s work on climate change has been hampered by its inability to interact with the growing and changing range of stakeholders involved in global health, while its health systems work also remains siloed.^16^ This is compounded by institutional reform weakness on issues of accountability, especially to external stakeholders, where changing challenges and stakeholder demands, for instance arising from climate change, have not led to any tangible change of institutional policies and systems over the past decade, quite different from the trajectory of other organizations.^17–19^ Also on a health policy level, the organization has been accused of inertia.^20^ More recently, WHO has begun to engage (very belatedly) with global climate change decision-making processes. At the UN Framework Convention on Climate Change 15th Conference of Parties (COP) (to the Kyoto Protocol) in Copenhagen in 2009, the WHO submitted a detailed paper entitled ‘Protecting the health of vulnerable people from humanitarian consequences of climate change and climate related disaster’. It also lobbied for the inclusion of health-relevant indicators to achieve ‘co-benefits’ at the June 2012 Earth Summit and at the 18th COP (Doha, December 2012) where it hosted a side-event on ‘Building sustainable health systems: Focus on Climate Resilience’,^21^ although it is unclear if any commitments from COP participants were achieved.

Yet despite these laudable attempts and initiatives, what is clearly missing is dedicated and visible leadership that coordinates the activities of different researchers, research bodies, policy makers and international organizations across relevant sectors including disaster management, climate and health care systems. WHO – the obvious leader on global health issues, as the only global institution dedicated to health – has not shown itself to be proactive in engaging with multiple cross-sector stakeholders on issues of global importance beyond its narrow health field. Yet, the nature of climate change means a global view beyond WHO’s traditional health sector comfort zone is essential. The World Bank has proved far better at understanding and promoting the connections between multiple stakeholders required for a response to climate change and sustainable development, although its actions on health have been more limited. Unless the WHO can significantly ramp up its engagement with, and traction in, international decision-making processes for sustainable development and climate change, the World Bank may consider it necessary to move to fill the gap on health and climate change, which it is well-positioned to do. Only through such leadership can health and health care systems’ development be effectively promoted in a post-MDG development framework that is defined by climate change. The cost of failure will be high.
